# When Attempting Chain Extension, Even Without Solvent, It Is Not Possible to Avoid Chojnowski Metathesis Giving D_3_

**DOI:** 10.3390/molecules26010231

**Published:** 2021-01-05

**Authors:** Mengchen Liao, Yang Chen, Michael A. Brook

**Affiliations:** Department of Chemistry and Chemical Biology, McMaster University, 1280 Main Street, W. Hamilton, ON L8S 4M1, Canada; liaom6@mcmaster.ca (M.L.); dychen@mcmaster.ca (Y.C.)

**Keywords:** silicone polymers, Piers–Chojnowski–Rubinsztajn–Kawakami reaction, chain extension by Si-H hydrolysis, siloxane metathesis, D_3_ synthesis

## Abstract

A simple, mild and efficient method to prepare HSi- or HOSi-telechelic, high-molecular-weight polydimethylsiloxane polymers (to 41,600 g·mol^−1^) using the one-shot hydrolysis of M^H^M^H^ is reported; titration of the water allowed for higher molecular weights (to 153,900 g·mol^−1^). The “living” character of the chain extension processes was demonstrated by adding a small portion of M^H^M^H^ and B(C_6_F_5_)_3_ (BCF) to a first formed polymer, which led to a ~2-fold, second growth in molecular weight. The heterogeneous reaction reached completion in less than 30 min, much less in some cases, regardless of whether it was performed neat or 50 wt% in dry toluene; homogeneous reactions in toluene were much slower. The process does not involve traditional redistribution, as judged by the low quantities (<3%) of D_4_ produced. However, it is not possible to avoid Chojnowski metathesis from M^H^DDM^H^ giving D_3_, which occurs competitively with chain extension.

## 1. Introduction

**Figure 1:**
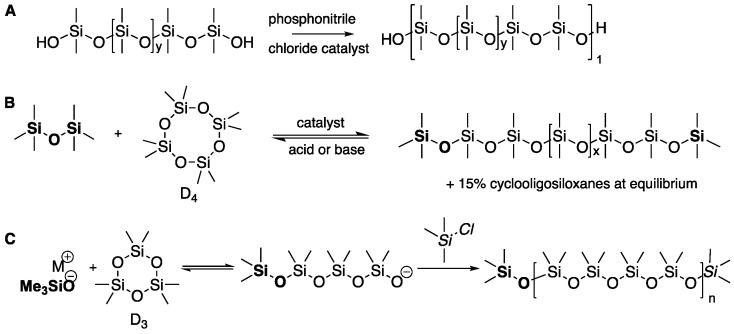
Traditional routes to silicone polymers: (**A**) silanol condensation; (**B**) redistribution; (**C**) ring-opening polymerization.

Polydimethylsiloxane (PDMS) oils are the parent commercial silicone polymers from which almost all other silicones are derived. PDMS possesses unique properties including low T_g_, good thermal stability, high optical transparency, excellent dielectric properties and excellent biocompatibility. Conventionally, two methods dominate the commercial preparation of high-molecular-weight PDMS oil: dehydration of silanol-terminated oligomers [1] and acid- or, particularly, base-catalyzed equilibration of cyclic monomers [2] (Figure 1A,B). However, each of these approaches suffers from inherent shortcomings. The former process, as with all condensation processes, slows down with increasing conversion, which makes the synthesis of high-molecular-weight polymers challenging; both processes typically lead to polymers with high dispersities *Đ*_M_. In the latter case, with base-catalyzed equilibration that has an equilibrium constant near 1, the desired polymer is accompanied by the formation of large quantities of cyclic monomers, e.g., <15% for D_4_ (D = ~Me_2_SiO~) [3,4] (Figure 1B). Cyclooligosiloxanes, particularly D_4_, have attracted different levels of concerns by regulatory agencies because of their purported environmental behaviors [5,6]; D_4_ concentrations are regulated in Canada and the UK [7,8]. Hence, there is an increasing consensus that the value of silicone polymers would be increased if they contained lower cyclooligosiloxane concentrations. Note that the removal of cyclic monomers from silicone oils becomes more difficult as the MW (molecular weight) and viscosities of both cyclics and oils increase.

The competing commercial process for synthesis of high-MW silicone oils is ring-opening polymerization [9], typically initiated by anions [10,11,12] (Figure 1C). Polymers with high MW can result, but the process is challenged by the need to be scrupulously dry to avoid premature termination; higher MW PDMS polymers with narrow dispersity *Đ*_M_ are more easily achieved when the more expensive, ring-strained monomer D_3_ is utilized as a starting material instead of D_4_.

Hydrosilane monomers, particularly HMeSiCl_2_ and HMe_2_SiCl, are produced in a direct process [13] in concentrations that typically exceed commercial needs. As a consequence, oligomers HMe_2_SiOSiMe_2_H) (M^H^M^H^) and polymers Me_3_Si(OSiMeH)_n_OSiMe_3_ (PHMS) based on these monomers are readily available. The compounds are potent reducing agents and, as Larson has noted, are both efficacious and inexpensive [14,15]. In organic synthesis, a benefit is the ease with which silicone products, after reduction, are readily separated from the desired organic product(s).

Tris(pentafluorophenyl)borane B(C_6_F_5_)_3_ (BCF), a metal-free, water-tolerant [16] and thermally stable (up to 270 °C) compound [17], is renowned for its effectiveness as a co-initiator for industrial olefin polymerization [18]. In his extensive and elegant studies of reduction of carbonyl groups, Piers showed that it was also a potent catalyst for hydrosilane reductions [17]. We used M^H^M^H^ in the presence of BCF to reduce the sulfur crosslinks in automobile rubber tires, permitting reuse of the organic constituents [19]. When using HSiEt_3_ in the presence of BCF, Piers et al. originally reported that over-reduction of carbonyl groups led first to silyl ethers and then to alkanes plus disiloxanes.

Rubinsztajn and Cella recognized that the Piers reduction was a new route to silicones, and that was first patented and then published in the open literature [20]. Chojnowski et al. have made several seminal contributions to our understanding of the mechanism of this process [21,22]. It is worth noting that growing optically active siloxanes from silanols, rather than alkoxysilanes, using B(C_6_F_5_)_3_ was pioneered by Kawakami [23]. In retrospect, perhaps we should have named the reaction the Piers–Chojnowski–Rubinsztajn–Kawakami (PCRK) reaction, rather than the PR reaction [24], and will do so for this paper.

**Figure 2:**
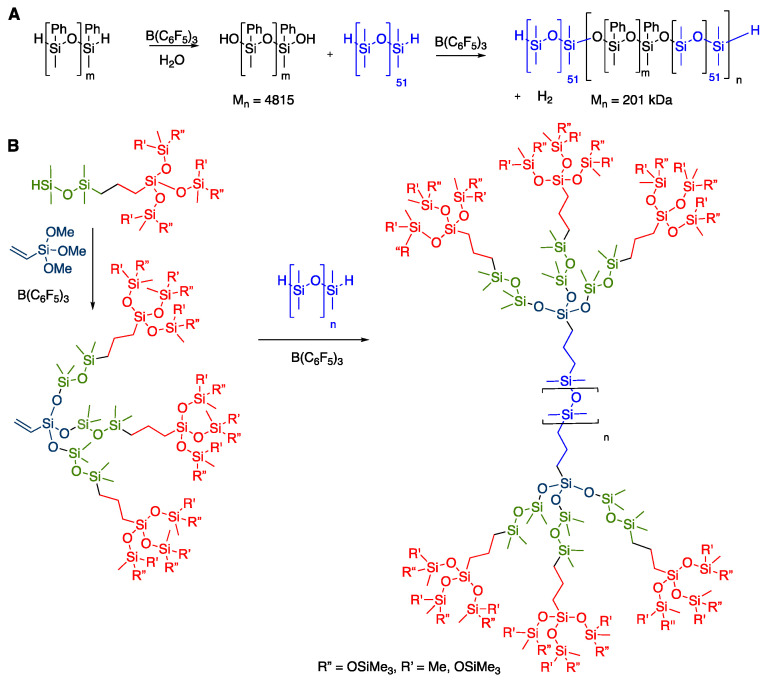
(**A**) Polymerization of telechelic HSi-silicones and water to give block copolymers. (**B**) Formation of highly branched silicones using the PCRK (Piers–Chojnowski–Rubinsztajn–Kawakami) reaction.

The PCRK reaction is a particularly convenient route to synthesize silicone polymers. One simply chooses the number of alkoxysilane or silanol substituents, or water, required for the synthesis of a given linear or branched monomer, and then adds the appropriate mono-, di- or oligofunctional HSi-containing molecules in the presence of BCF [25]. It is thus possible to create linear polymers, including block-copolymers, simply by combining telechelic HSi + HOSi silicones or HSi silicones + water [26,27,28]. We have previously exploited this method to reliably introduce branches along linear silicone backbones [29] including, in the limit, highly branched dendron-like structures [30], including MDTQ resins (D = Me_2_SiO_2/2_) [31] (Figure 2).

**Figure 3:**
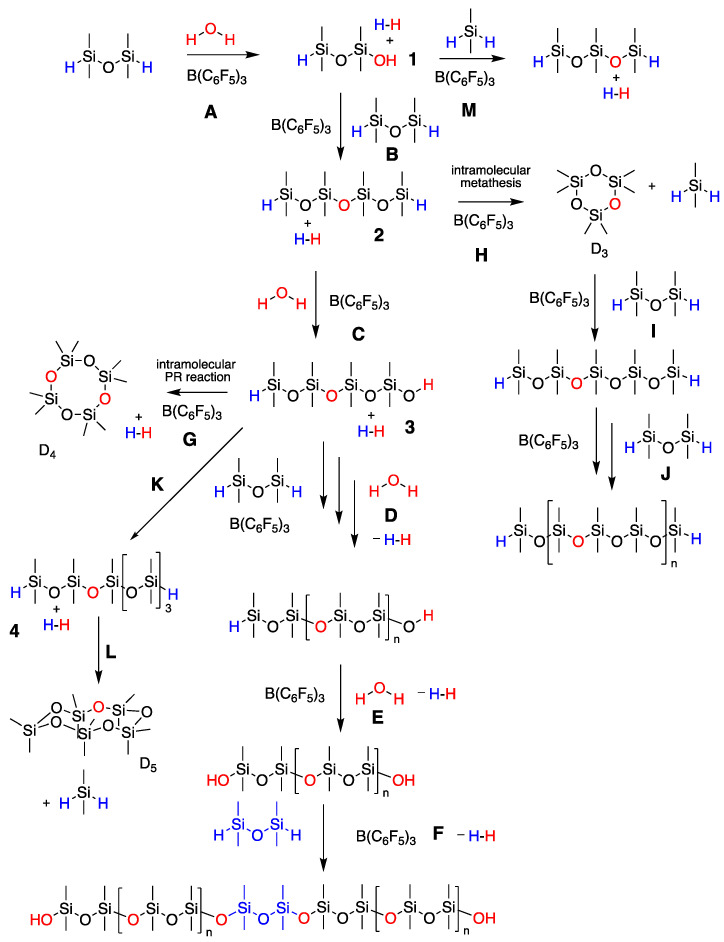
Proposed reactions for cyclooligosiloxane and polymer formation from M^H^M^H^. Hydrolysis of SiH compounds to give hydroxy-capped (**A**) dimer; (**C**) tetramer; (**D**,**E**) polymers. Chain extension to give HSi-terminated (**B**) tetramer; (**F**) polymer; (**K**) hexamer. Chojnowski metathesis to give (**H**) D_3_; (**K**) D_5_; (**L**) Me_2_SiH_2_; (**G**) Cyclization to give D4. (**I**,**J**). Metathesis polymerization to higher polymers. (**M**) Chain homologation between silanols and Me_2_SiH_2_.

Tetramethyldisiloxane (M^H^M^H^) is an inexpensive, atom-efficient hydrosilane that has been selected as a silane source for a number of reactions [14,32,33]. We note that, at the time of writing, M^H^M^H^ is slightly more expensive that non-functional D_3_ and much more expensive than D_4_ monomers. Attempts to produce high-molecular-weight PDMS oil from M^H^M^H^ and H_2_O in aqueous media, a traditional PCRK reaction (mole ratio, [OH]/[SiH] = 56) was made by the group of Ganachaud who reported formation of an elastomer; it was concluded that the reaction was not readily controllable [32]. Chojnowski et al. showed under anhydrous Schlenk line conditions that the oligomerization of M^H^M^H^ (Figure 3H–J) in the presence of a BCF catalyst led to HSi-terminated oligomers and D_3_—Chojnowski metathesis [22], however, with large quantities of D_3_ that were produced M^H^DDM^H^; secondary copolymerization of D_3_ and M^H^M^H^ with activation with B(C_6_F_5_)_3_ could be used to lead to higher molecular weight polymers [33].

Neither the Ganachaud nor Chojnowski outcomes with M^H^M^H^ matched our experience of HSi-terminated silicones in the presence of water and B(C_6_F_5_)_3_, which led smoothly to high-molecular-weight PDMS oils [26] (Figure 2). We hypothesized that differences arose because of the quantity of available water and BCF and, perhaps, other experimental conditions. Herein, we report a simple and mild process for the formation of HOSi- or HSi-terminated high-molecular-weight PDMS oil by the hydrolysis of M^H^M^H^ in a kinetic process that generates relatively low quantities of D_4_ (<3%). However, dilution with good solvents for silicone enhances the fraction of D_3_ produced alongside the polymer.

## 2. Results

The reaction between M^H^M^H^ and water leads first to disiloxanol **1** and then to the tetrasiloxane formation **2** (Figure 3A,B); both reactions are rapid and lead to the concomitant formation of hydrogen at the water droplet interface. *Note: caution must be taken, as this can lead to a pressure spike of this flammable gas*. Repetition of these processes lead to PDMS polymer initially terminated with SiH and then SiOH groups (Figure 3C–E). The processes were followed using ^1^H-NMR (particularly the relationship between integrated peak areas of Si-*H* (~4.7 ppm vs. Si-C*H*_3_ ~0.1–0.2 ppm [34,35])) and ^29^Si-NMR spectra that allow clear differentiation between cyclic silicone monomers, linear silicone polymers and HSi-terminated linear materials [35], as well as gel permeation chromatography (GPC) and Fourier-transform infrared spectroscopy (FTIR; Supplementary Material, SM).

The objective of the research was to identify simple, efficient processes that would lead to linear PDMS, optionally terminated with SiH groups. Preliminary experiments examined the impact of reaction parameters, including catalyst concentration, solvent and, in particular, the effect of homogeneity between water and silicone phases during the course of hydrolysis. In addition, the impact of “one-shot” addition of reagents was compared to a titration in which water and/or additional BCF were added when reaction ceased.

Simply mixing M^H^M^H^, B(C_6_F_5_)_3_ stock solution in dry toluene with bulk water led to linear silicones (entries 1–3, Table 1). Unsurprisingly, reactions were faster with more catalyst, but satisfactory rates were already achieved with only 0.02 mol% of this not inexpensive catalyst (<30 min, see below). Adding water in excess to the stoichiometric requirement did not lead to an improved outcome: cyclics were formed as a byproduct (see below, Supplementary Materials). Dilution with solvent, initially dichloromethane (containing 72.5 ppm water), demonstrated that much higher molecular weight polymers were accessible with even lower levels of catalyst (entries 4–6, Table 1); however, D_4_ was also a byproduct of these reactions (Supplementary Materials). Thus, if high molecular weight is most desired, using small amounts of organic solvents is beneficial (entries 5, 6, Table 1); if suppression of cyclics is key, use slightly more BCF (entry 1, Table 1). Note that the use of a completely homogeneous reaction was disadvantageous for practical and kinetic reasons. For example, the reaction of 1 g of M^H^M^H^ would require 14.3 mL of “wet” toluene (saturated with sufficient water to complete SiH hydrolysis), so scale up would not be practicable. When we did attempt the reaction on a small scale under these conditions, it proceeded for only a small extent over 5 h and then remained unchanged even after 24 h (see Supplementary Materials).

### 2.1. High-Molecular-Weight PDMS Preparation Using Hydrolysis

The preparation of telechelic PDMS polymers from M^H^M^H^ terminated with either SiOH or SiH groups was straightforward by adding water in a one-shot process in the presence of 0.02% B(C_6_F_5_)_3_. The two-phase reaction of water/silicone was surprisingly rapid; reaction times of less than 30 min led to polymers and concomitant formation of H_2_. The hydrolysis/condensation reactions in 50 wt% toluene were yet more rapid, as judged by the rate of build of the polymer molecular weight (entries 1–9, Table 2). We have not determined if the hydrolytic processes in toluene/water involve only homogeneous or a mixture of homogeneous/heterogeneous steps. The efficiency of chain extension will decrease as the living polymer increases in size, particularly above the entanglement limit of about 29,000 g·mol^−1^ (note: in the literature, reported entanglement limits ranges from about 15,000–35,000 g·mol^−1^. Here we use data from the seminal study of Mrozek et al. [36]). This was clearly observed here, as the final M_n_ were 31–45 kg·mol^−1^ (entry 9, Table 2); complete consumption of SiH groups at higher conversion terminates polymerization by forming HOSi-terminated polymers. Re-initiation of such “dead” polymers is straightforward; however, addition of small quantities of M^H^M^H^ caps the SiOH groups leading to dimerization or higher homologues of the existing polymer (entry 10, Table 2, Figure 3F). That is, a beneficial consequence of this process is that if HSi-terminated polymers are desired, one need only add excess M^H^M^H^ to cap (Silicone-Me_2_SiOH → Silicone-(Me_2_SiO)_2_Me_2_SiH) and, if desired, grow the polymers before quenching the catalyst. This observation demonstrates that the process is living (entry 10, Table 2) [26]. Note that any residual water will compete for the silanol chain ends such that, if high molecular weights are desired, it is advantageous before capping to remove using distillation the small quantities of low molecular weight materials present, including residual water.

### 2.2. Managing Cyclics

Our objective was to develop simple, practicable polymer syntheses that avoided the need for inert gas blankets; a septum with a bubbler was used to control pressure. One of the challenges presented by M^H^M^H^ is its high volatility, which was problematic with or without solvents. The evolution of cyclics was followed by a combination of gravimetric analysis for volatile products (trapped in a cold trap that permitted cogenerated H_2_ to escape) and, for the polymerization mixture, ^29^Si-NMR, which is particularly sensitive to subtle differences in the chemical environment of D units; it is straightforward to distinguish D_3_ (−8.3 ppm), D_4_ (−19.1 ppm) and D_5_ (−21.5 ppm) from D units in linear polymers (−21.6 ppm) [37]. M^H^M^H^ was the main constituent captured in a cold finger, with small amounts of M^H^DM^H^, Me_2_SiH_2_ and D_3_ (entries 7,8, Table 1).

**Figure 4:**
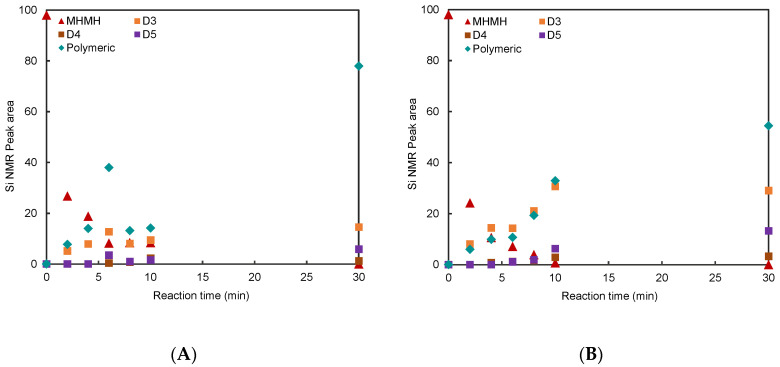
(**A**) Conversion to polymer without solvent. (**B**) Conversion to polymer in 50 wt% toluene. Rate of reaction (loss of HSi) during hydrolysis of M^H^M^H^ was monitored using ^1^H-NMR and silicone constituents using ^29^Si-NMR. Integrations assumed identical sensitivity for all D units and are normalized to 100%.

The hydrolysis/condensation of M^H^M^H^ in dilute, homogenous toluene solution was very slow. However, two-phase reactions (water/silicone or water/silicone+toluene) were very rapid reactions and completed in <30 min. Reactions can occur at the interface or within the organic fluid. In the case of the water/silicone mixture, the polymer yield was near 80%, with competing growth in D_3_ and small amounts of D_5_; D_4_ was only inefficiently formed (Figure 4A). By contrast, even with the small dilution provided in the 50% water/silicone+toluene system, much less polymer was formed at the expense of D_3_ and D_5_ production, again with little D_4_ (Figure 4B).

Cyclic monomers are important starting materials for silicone synthesis. As noted above, the ring strain in D_3_ makes it an attractive starting material for ring-opening polymerizations. On the other hand, there is an interest for a variety of reasons in making cyclic-free silicones, particularly from an inexpensive starting material like M^H^M^H^ that polymerizes so rapidly. We had hoped to be able to fine tune the polymerization to create high molecular polymers in the absence of cyclics.

After dimerization, M^H^M^H^ → M^H^DDM^H^ **2**, the tetramer can undergo hydrolysis to give **3** then further chain extension, or cyclization to give D_4_ (Figure 3D vs. Figure 3G). The generation of D_4_ during polymerization under equilibrating conditions is favored both enthalpically and entropically, possessing virtually zero ring strain, and the SiO bond strength in cyclics is similar to those in linear chains and larger number of molecules than in linear polymers [1]. In neither of the cases examined was D_4_ a significant product (Figure 4, Table 1). Thus, under these conditions of chain extension, **3** outcompetes cyclization (Figure 3D,K vs. Figure 3G). Adding small amounts of toluene led to an increase in D_4_ product only from 1% to 3%.

More problematic, with respect to cyclics, was the formation of D_3_ and, to a lesser extent, D_5_. The most concentrated (neat) solution produced 13% D_3_, but in the diluted sample 33% of the product mixture was D_3_, in addition to linear polymer (entries 7,8, Table 1). These data show that, even in the presence of water, the Chojnowski metathesis to give D_3_ and Me_2_SiH_2_ from **2** is highly competitive with hydrolysis (Figure 3H). The presence of D_5_ in such high quantities is consistent with chain extension from **3** to **4** and then a different Chojnowski metathesis leading to D_5_ and Me_2_SiH_2_ (e.g., Figure 3K,L. We thank a referee for this suggestion). It is believed that the formation of M^H^DM^H^, found in the cold trap, can be ascribed to reactions with Me_2_SiH_2_ with disiloxanes in the presence of B(C_6_F_5_)_3_ (Figure 3M).

Previous experience with polymer chain extension of HSi-telechelic polymers with water did not lead to the formation of cyclics. Under these conditions with low catalyst concentrations (0.02%) (BCF/H_2_O and B(C_6_F_5_)_3_∙OH_2_ [38]), redistribution reactions are similarly not efficient. While the reactions here were complete in 30 min, there was no change in the cyclics profile between 30–180 min (SM); redistribution would favor the formation of D_4_ which, of the cyclics characterized, was formed in the lowest concentration. Under equilibration in the absence of solvents, the normal concentration of D_4_ is ~15% [4]. Similarly, redistribution cannot explain the higher concentrations of D_3_ and D_5_.

The formation of cyclics vs. linears is therefore a consequence of the kinetics of intra- vs. intermolecular reactions within a silicone/organic solvent, or at the water interface. When done in 50wt% toluene, more cyclics, particularly D_3_, were produced than when the only solvent for the silicone was M^H^M^H^ and silicone products themselves, i.e., than when the system was more concentrated (Figure 4B vs. Figure 4A). This shows the power of Chojnowski metathesis. The conversion of **2** → D_3_ (and **4** → D_5_) effectively competes with hydrolysis of **2** → **3** when the reaction is slightly diluted, and is an important reaction even when done neat. The formation and then disappearance in the ^29^Si-NMR of a maximum of 1.4% of Me_2_SiH_2_ was observed based on D-units (for full details of all intermediates from Figure 4, see Supplementary Materials). M^H^DM^H^, observed in the cold trap, could arise from reaction of Me_2_SiH_2_ with **3** and there may be additional homologation reactions that consume this reactive material. We cannot distinguish between polymerization involving hydrolysis/condensation (Figure 3B–E) vs. the reaction between D_3_ and hydrosiloxanes, as shown by Chojnowski et al. (Figure 3I,J) [33]. However, the rapid rate and high molecular weight support the hydrolytic/condensation process as dominant.

Molecular weight is, of course, an important determinant of polymer properties. One advantage of this process is the relatively narrow dispersities achieved in the higher molecular weight polymers (Table 2). They do not match dispersities of polymers formed from ring-opening polymerization of D_3_ but, apart from the challenge of managing hydrogen generation, are easier to perform. In part, the objectives of the work were met. It was possible to obtain rapid polymerization of M^H^M^H^ with water to get medium-molecular-weight linear silicones terminated, if desired, with SiH groups. High concentrations facilitated the rates of polymerization. The process is living and any premature suppression of polymerization by complete hydrolysis to SiOH groups can be overcome by the addition of small amounts of M^H^M^H^, preferably after removing any residual water, a process that allows much longer polymers to form. However, unlike the hydrolytic process with starting materials that have a higher MW (e.g., DP > 6) [26], polymerization competed with undesired cyclic formation. Distinctions between these in previous studies are mostly related to concentrations. The important precedent work of Ganachaud showed that with very high water and higher catalyst concentrations, complex equilibration reactions occur under less controlled conditions [32]. On the other hand, with exceptionally low concentrations of water and catalysts, Chojnowski et al. showed metathesis of **2** dominates the process; one can elect to capture and then use D_3_ for polymerization [21,22]. This work demonstrates the power of Chojnowski metathesis. Even without a solvent, efficient formation of D_3_ is observed, up to 33%, made worse even by small amounts of solvent.

## 3. Experimental Section

### 3.1. Materials

Tetramethyldisiloxane (M^H^M^H^) was purchased from Gelest (Morrisville, PA, USA) and dried over molecular sieves (obtained from Sigma Aldrich (Oakville, ON, Canada), Molecular sieves, 4 Å beads, 8–12 mesh) before use. Ultrapure water (18 MΩ-cm) was obtained from Barnstead Easy pure RF (https://www.barnstead-water.com/). The B(C_6_F_5_)_3_ catalyst was provided by Alfa Aesar (Tewksbury, MA, USA). Toluene (Caledon, ON, Canada) was dried over an activated alumina column (2.3 ppm water present). “Wet” DCM (72.5 ppm water present) and “wet” toluene (466.6 ppm water present) were purchased from Caledon (Georgetown, ON, Canada).

### 3.2. Methods

^1^H- and ^29^Si-NMR spectra were recorded on a Bruker Advance 600 MHz nuclear magnetic resonance spectrometer using deuterated solvent chloroform-*d* (Milton, ON, Canada). Chromium(III) acetylacetonate (3 × 10^−3^ mol·L^−1^) was added as a relaxation reagent for some of these measurements.

Gel permeation chromatography was carried out using a Viscotek GPC Max (VE 2001 GPC Solvent/Sample Module) (no longer manufactured). The system was equipped with a Viscotek VE 3580 RI Detector, a Viscotek 270 Dual Detector, and a PolyAnalytik SupeRes PAS-101 (8 mm Å~30 cm) column with a single pore, styrene-divinylbenzene gel, 6 μm particle size (London, Canada). It was additionally calibrated using a single narrow polydispersity polystyrene standard (93 kDa) from Polymer Laboratories. Toluene was used as the eluent at a flow rate of 1.0 mL·min^−1^.

FTIR data were collected on a Nicolet 6700 FTIR using Thermo Electron’s OMNIC software (version 8.0).

GC-MS analyses were performed using an Agilent 6890 N gas chromatograph (Santa Clara, CA, USA) equipped with a DB-17ht column (30 m × 0.25 mm i.d. × 0.15 μm film, J & W Scientific), and coupled to an Agilent 5973 MSD single quadruple mass spectrometer (Santa Clara, CA, USA). One microliter of sample was injected using Agilent 7683 autosampler (Santa Clara, CA, USA) using an injector temperature was 250 °C and a carrier gas (helium) flow of 0.8 mL/min. The transfer line was set to 280 °C and the MS source temperature was 230 °C. The column temperature started at 40 °C, raised to 70 °C at 5 °C/min, raised to 95 °C at 10 °C/min, raised to 300 °C at 40 °C/min, and was then held at 300 °C for 8 min for a total run time of 21.73 min. Full scan mass spectra between m/z 50 and 800 were acquired, with the MS detector turned off between 2.0–2.8 min for solvent. Please note that the sample is dissolved in propyl formate.

Water concentrations were determined using Karl Fischer titrations (Mettler Toledo DL39 Coulometer) (Columbus, OH, USA) with a one-component system containing a Hydranal Composite solution.

### 3.3. Experimental Procedures for High-Molecular-Weight PDMS Preparation Using Hydrolysis

The following paragraphs refer to entries in Table 1.

#### 3.3.1. One-Shot Addition

B(C_6_F_5_)_3_ stock solution in dry toluene.

To a pre-dried 25.0 mL vial added B(C_6_F_5_)_3_ (0.052 g, 0.01 mmol) catalyst together with dry toluene (1.0156 mL, 0.881 g) to prepare a B(C_6_F_5_)_3_ stock solution in dry toluene (0.01 M).

entry 1 ([BCF]/[SiH] = 0.02 mol%; [OH]/[SiH] = 1; one-shot) (Table 1):

To a pre-dried 100.0 mL round-bottomed flask we added tetramethyldisiloxane (M^H^M^H^) (134 g·mol^−1^, 3.13 g, 0.02 mol) and distilled water (18 g·mol^−1^, 0.402 mL, 0.02 mol) which was then capped with a septum with a needle with a bubbler open to atmosphere to balance the pressure. The mixture was stirred for 5–10 min prior to the addition of B(C_6_F_5_)_3_ stock solution in dry toluene (0.01 M, 0.094 mL, 0.0094 mmol). The B(C_6_F_5_)_3_ stock solution was added by an Eppendorf pipette into the flask through opening the septa. Once the B(C_6_F_5_)_3_ stock solution was added, immediately, vigorous bubbling inside of the round bottle flask was observed. The mixture was stirred at room temperature for 3 h and the reaction was quenched by alumina for 5 h and the residual water droplet was removed by adding sodium sulfate.

entry 2 ([BCF]/[SiH] = 0.02 mol%; [OH]/[SiH] = 3.2; one-shot) (Table 1):

To a pre-dried 100.0 mL round-bottomed flask we added tetramethyldisiloxane (M^H^M^H^) (134 g·mol^−1^, 5.01 g, 0.037 mol) and distilled water (18 g·mol^−1^, 2.144 mL, 0.119 mol) which was then capped with a septum with a needle with a bubbler open to atmosphere to balance the pressure. The mixture was stirred for 5–10 min prior to the addition of B(C_6_F_5_)_3_ stock solution in dry toluene (0.01 M, 0.149 mL, 0.0149 mmol). The B(C_6_F_5_)_3_ stock solution was added by an Eppendorf pipette into the flask through opening the septa. Once the B(C_6_F_5_)_3_ stock solution was added, immediately, vigorous bubbling inside of the round bottle flask was observed. The mixture was stirred at room temperature for 3 h and the reaction was quenched by alumina for 5 h and the residual water droplet was removed by adding sodium sulfate.

entry 3 ([BCF]/[SiH] = 0.1 mol%; [OH]/[SiH] = 1; one-shot) (Table 1):

To a pre-dried 100.0 mL round-bottomed flask we added tetramethyldisiloxane (M^H^M^H^) (134 g·mol^−1^, 1.00 g, 0.0074 mol) and distilled water (18 g·mol^−1^, 0.134 mL, 0.0074 mol) which was then capped with a septum with a needle with a bubbler open to atmosphere to balance the pressure. The mixture was stirred for 5–10 min prior to the addition of B(C_6_F_5_)_3_ stock solution in dry toluene (0.01 M, 0.150 mL, 0.015 mmol). The B(C_6_F_5_)_3_ stock solution was added by an Eppendorf pipette into the flask through opening the septa. Once the B(C_6_F_5_)_3_ stock solution was added, immediately, vigorous bubbling inside of the round bottle flask was observed. The mixture was stirred at room temperature for 3 h and the reaction was quenched by alumina for 5 h and the residual water droplet was removed by adding sodium sulfate. The mixture was stirred at room temperature for 3 h and the reaction was quenched by alumina. The product was collected by filtration through Celite under reduced pressure.

#### 3.3.2. Portion by Portion Addition

entry 4 (overall [BCF]/[SiH] = 0.008 mol%; [OH]/[SiH] = 1; portion by portion adding of B(C_6_F_5_)_3_ catalyst, neat) (Table 1):

To a pre-dried 100.0 mL round-bottomed flask we added tetramethyldisiloxane (M^H^M^H^) (134 g·mol^−1^, 5.01 g, 0.037 mol) and the distilled water (18 g·mol^−1^, 0.67 mL, 0.037 mol) which was then capped with a septum with a needle with a bubbler open to atmosphere to balance the pressure. The mixture was stirred for 5–10 min prior to the addition of B(C_6_F_5_)_3_ stock solution in dry toluene (0.001 M, 0.149 mL, 0.00149 mmol, [BCF]/[SiH] = 0.002 mol%, first portion of catalyst). The B(C_6_F_5_)_3_ stock solution was added by an Eppendorf pipette into the flask through opening the septa. Bubbles immediately formed once the catalyst was added. After no bubble formation was observed (throughout the reaction, water droplets always existed, indicating sufficient amount of water), once again, B(C_6_F_5_)_3_ stock solution in dry toluene (0.001 M, 0.149 mL, 0.00149 mmol, [BCF]/[SiH] = 0.002 mol%, second portion of catalyst) was added and vigorous bubbling occurred again. The procedure of adding B(C_6_F_5_)_3_ stock solution in dry toluene (0.001 M, 0.149 mL, 0.00149 mmol, [BCF]/[SiH] = 0.002 mol%) repeated until no bubbles were observed even after the addition of a fresh portion of catalyst; overall, four portions of catalyst were added (0.008 mol%). The mixture was stirred at room temperature for 22 h and the reaction was quenched by alumina for 5 h and the residual water droplet was removed by adding sodium sulfate.

entry 5 (overall [BCF]/[SiH] = 0.006 mol%; [OH]/[SiH] = 0.73; portion by portion, 1.3 g·mL^−1^, mass of M^H^M^H^ versus volume of DCM) (Table 1):

To a pre-dried 100.0 mL round-bottomed flask we added tetramethyldisiloxane (M^H^M^H^) (134 g·mol^−1^, 4.12 g, 0.031 mol) and “wet” DCM (3 mL, 72.5 ppm water, 0.016 mmol of water present) which was then capped with a septum with a needle with a bubbler open to atmosphere to balance the pressure. The mixture was stirred for 5–10 min prior to the addition of B(C_6_F_5_)_3_ stock solution in dry toluene (0.001 M, 0.119 mL, 0.0012 mmol, [BCF]/[SiH] = 0.002 mol%, first portion of catalyst) followed by a quick addition of the distilled water (18 g·mol^−1^, 0.074 mL, 0.0041 mol, [OH]/[SiH] = 0.13, first portion). The B(C_6_F_5_)_3_ stock solution and water were added by an Eppendorf pipette into the flask through opening the septa and a vigorous bubble formation was observed immediately after the addition of water. Here is how we decided to whether add catalyst or water: First, we added the distilled water (18 g·mol^−1^, 0.055 mL, 0.0031 mol, [OH]/[SiH] = 0.1, second portion). (i) If bubble formation was observed, the indicated the cease of bubbles originated from insufficient amount of water. (ii) If no bubble formation was observed after the addition of the water indicating the catalyst was insufficient, then B(C_6_F_5_)_3_ stock solution in dry toluene (0.001 M, 0.119 mL, 0.0012 mmol, [BCF]/[SiH] = 0.002 mol%) was added. The above-mentioned procedure repeated until no bubbles formed regardless of the addition of catalyst or water; overall, [BCF]/[SiH] = 0.006 mol%; [OH]/[SiH] = 0.73. The mixture was stirred at room temperature for 5.6 h and the reaction was quenched by alumina for 5 h and the residual water droplet was removed by adding sodium sulfate.

entry 6 (overall [BCF]/[SiH] = 0.004 mol%; [OH]/[SiH] = 0.75; portion by portion, 1.0 g·mL^−1^, mass of MHMH versus volume of DCM) (Table 1):

To a pre-dried 100.0 mL round-bottomed flask we added tetramethyldisiloxane (M^H^M^H^) (134 g·mol^−1^, 3.05 g, 0.023 mol) and “wet” DCM (3 mL, 72.5 ppm water, 0.016 mmol of water present) which was then capped with a septum with a needle with a bubbler open to the atmosphere to balance the pressure. The mixture was stirred for 5–10 min prior to the addition of B(C_6_F_5_)_3_ stock solution in dry toluene (0.001 M, 0.092 mL, 0.00092 mmol, [BCF]/[SiH] = 0.002 mol%, first portion of catalyst) followed by a quick addition of the distilled water (18 g·mol^−1^, 0.062 mL, 0.0034 mol, [OH]/[SiH] = 0.15, first portion). The B(C_6_F_5_)_3_ stock solution and water were added by an Eppendorf pipette into the flask through opening the septa and a vigorous bubble formation was observed immediately after the addition of water. Here is how we decided to whether add catalyst or water: First, we added the distilled water (18 g·mol^−1^, 0.041 mL, 0.0023 mol, [OH]/[SiH] = 0.1, second portion). If bubble formation was observed, this indicated the cessation of bubbles originated from an insufficient amount of water. If no bubble formation was observed after the addition of the water, indicating the catalyst was insufficient, then B(C_6_F_5_)_3_ stock solution in dry toluene (0.001 M, 0.092 mL, 0.00092 mmol, [BCF]/[SiH] = 0.002 mol%) was added. The above-mentioned procedure was repeated until no bubbles formed regardless of the addition of catalyst or water; overall, [BCF]/[SiH] = 0.004 mol%; [OH]/[SiH] = 0.75. The mixture was stirred at room temperature for 2.6 h and the reaction was quenched by alumina for 5 h and the residual water droplet was removed by adding sodium sulfate.

#### 3.3.3. Capturing the Volatiles, Entries 7, 8

To a pre-dried 100.0 mL round-bottomed flask, tetramethyldisiloxane (M^H^M^H^) (134 g·mol^−1^, 10.38 g, 77.5 mmol) with distilled water (18 g·mol^−1^, 1.34 mL, 74.4 mmol)(entry 7, Table 1) was added. To the mixture B(C_6_F_5_)_3_ stock solution in dry toluene (0.1 M, 0.298 mL, 0.0298 mmol) was added. The cold trap condenser was submerged in dry ice/acetone with the addition of acetone and was directly connected to the round-bottomed flask through 6 mm O.D. Pyrex tubing. The mixture in the round bottle flask was stirred at room temperature for 3h and the reaction was quenched by alumina and the residual water droplet was removed by adding sodium sulfate with 5h quenching time. Before the work up procedures, the mixtures in the round bottle flask were weighed and characterized by ^1^H-, ^29^Si-NMR (SR) followed by filtration through Celite under reduced pressure. The same process was repeated for the experiment that also included toluene (entry 8, Table 1): M^H^M^H^ (134 g·mol^−1^, 10.32 g, 77.0 mmol) with distilled water (18 g·mol^−1^, 1.34 mL, 74.4 mmol), dry toluene (6.73 mL, 7.76 g) and B(C_6_F_5_)_3_ stock solution in dry toluene (0.1 M, 0.298 mL, 0.0298 mmol).

### 3.4. Experimental Procedure for Controlled Growth of Linear PDMS Using M^H^M^H^ and Water

The following paragraphs refer to Table 2.

#### 3.4.1. In Neat Water, or in Water/Toluene Mixtures

To a series of pre-dried 25.0 mL vials we added M^H^M^H^ (134 g·mol^−1^, 1.04 g, 7.8 mmol) with distilled water (18 g·mol^−1^, 0.134 mL, 7.4 mmol). To the mixtures, B(C_6_F_5_)_3_ stock solution in dry toluene (0.1 M, 0.03 mL, 0.003 mmol, respectively) was added and stirred at room temperature. At time points from 2–180 min alumina was added to quench the reaction at different time intervals together with ~0.5 g of sodium sulfate to remove the residual water. Before the work up procedures, the products were characterized by ^1^H-, ^29^Si-NMR (SR) followed by filtration through Celite under reduced pressure. The same process was repeated for the experiments that also included toluene: M^H^M^H^ (134 g·mol^−1^, 1.04 g, 7.8 mmol) with distilled water (18 g·mol^−1^, 0.134 mL, 7.4 mmol), dry toluene (0.677 mL, 0.585 g) and B(C_6_F_5_)_3_ stock solution in dry toluene (0.1 M, 0.03 mL, 0.003 mmol), respectively, the mixture was then stirred at room temperature (entries 1–9, Table 2).

#### 3.4.2. Chain Extension

To a 10.0 mL round-bottomed flask we added tetramethyldisiloxane (M^H^M^H^) (134 g·mol^−1^, 0.015 mL, 0.011 g, 0.085 mmol) with pre-prepared PDMS oil terminated with silanol (21,300 g·mol^−1^, 0.90 g, 0.042 mmol, entry 4, Table 1). To be noted here, to the pre-prepared silanol PDMS we performed Kugelrohr distillation at 100 °C under vacuum (635 mmHg) for 60 min before running the GPC to remove the residual water and volatile small molecules. To the mixture, B(C_6_F_5_)_3_ stock solution in dry toluene (0.01 M, 0.0086 mL, 0.086 µmol) was added and stirred at room temperature. After 3 h, alumina was added to the mixture to quench the reaction. The product was collected by filtration through Celite under reduced pressure (Entry 10, Table 2).

## 4. Conclusions

The simple, efficient and mild process of hydrolyzing M^H^M^H^ leads to high-molecular-weight PDMS (maximum 153,900 g·mol^−1^) in a controllable and “living” manner. The reactions can be completed in as short a time as 30 min, in two-phase reactions using either bulk water with neat M^H^M^H^ or M^H^M^H^ in dry toluene (50 wt%). Under either set of conditions, however, low D_4_ (1–3%) content is produced and higher values of D_5_ (2–10%) and particularly D_3_ (13–33%) are produced. These outcomes are attributed to the particular efficiency of Chojnowski metathesis in organic solvents, even with high water content.

## Figures and Tables

**Table 1:**
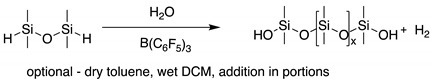
High-molecular-weight PDMS (polydimethylsiloxane) preparation using hydrolysis.

Entry ^a^	[BCF]/ [SiH] mol%	[OH]/ [SiH] ^a^	Addition ^b^	M^H^M^H^/DCM ^c^ (g·mL^−1^)	Cyclics % ^d^	PDMS % ^d^	M_n_ (g·mol^−1^)	M_w_ (g·mol^−1^)	*Đ*_M_	Mass Bal ^e^
1	0.1	1	O	-	2	98	43,700	129,200	2.97	70.1
2	0.02	1	O	-	26	74	52,700	105,400	2.00	71.3
3	0.02	3.2	O	-	5.9	94	41,600	101,500	2.44	70.6
4	0.008	1	P	-	5.9	92	20,700	32,500	1.57	79.3
5	0.006	0.73	P	1.3	12.9	87	111,400	221,500	1.99	95.6
6	0.004	0.75	P	1	5.7	94	152,500	314,000	2.06	82.9
**M^H^M^H^/PhCH_3_^c^ (g·mL^−1^)**										
7	0.02	1	O	-	18.9	81.2	20,500	37,400	1.82	73.8 ^f^
8	0.02	1	O	1.53	47.9	52.1	40,000	63,100	1.58	89.1 ^g^

^a^ Stoichiometric addition of bulk water except for entries 5, 6. Reactions were quenched between 2.5 and 22 h (SM). Most reactions were complete within 30 min (see below), conditions under which the catalyst remains highly effective [16]. ^b^ O = one-shot reaction; P = reagents were added portion by portion, each time bubble evolution ceased. ^c^ DCM contained 72.5 ppm water. Toluene was dry. Note: experiments in Table 1 were completed in DCM to optimize reaction conditions. We then switched to the greener solvent toluene. ^d^ Fraction of D units in D_3_ + D_4_ + D_5_ or PDMS based on integration in ^29^Si-NMR. ^e^ Mass % of non-volatiles. ^f^ Cold trap (volatiles) contained 23.9% (total mass balance 97.7%), which consisted of M^H^M^H^ 50%, Me_2_SiH_2_ 42%. ^g^ Cold trap contained 10.5% (total mass balance 99.6%), which consisted of M^H^M^H^ 66%, Me_2_SiH_2_ 26%.

**Table 2:**
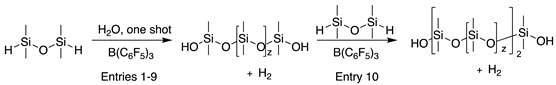
Molecular weight versus reaction time using hydrolysis of M^H^M^H^.

Neat						H_2_O/Toluene ^a^			
Entry	Time(min)	M_n_	MW	*Đ*_M_	MW ^b^	M_n_	MW	*Đ*_M_	MW ^b^
1	0	-	-	-	134	-	-	-	134
2	2	-	-	-	240	-	-	-	250
3	4	-	-	-	300	-	-	-	310
4	6	-	-	-	520 ^c^	-	-	-	330
5	8	-	-	-	310	-	-	-	440
6	10	-	-	-	330	-	-	-	1030
7	30	21,600	54,000	2.50	-	39,000	69,700	1.79	-
8	60	21,800	63,000	2.90	- ^a^	45,300	97,100	2.14	-
9	180	31,200	82,900	2.66	- ^a^	45,500	79,900	1.76	-
		Chain	Extension ^d^						
		Starting	Polymer			Product	Polymer		
10		21,300	33,200	1.56	→	41,900	137,000	3.27	

^a^ Reactions performed with 50 wt% M^H^M^H^ dry toluene + liquid water. ^b^ Calculated based on Si*H* peak area versus SiC*H*_3_ (integrated to 100) region in the ^1^H-NMR. ^c^ The observation at 6 min. is considered an outlier. ^d^ Starting polymer entry 4, Table 1 contained no cyclics, and none formed by ^29^Si-NMR; mass balance 99.9%.

## Data Availability

Data is contained within the article or supplementary material.

## Supplementary Materials

Figures S1 and S2: kinetic plots of reaction progress with and without toluene solvent, ^1^H-NMR and ^29^Si-NMR and IR characterization of each datapoint on Figure S1, Tables S1 and S2, GC and NMR data for the cold trap with and without toluene solvent (entries 7, 8, Table 1), NMR data for polymer (entry 10, Table 2).

## Dedication

Julian Chojnowski is a father of modern silicone polymer chemistry. His scientific prowess and excellence is matched by his generosity. When I asked to use his name in what became the “Piers–Rubinsztajn reaction”, he wanted instead to have his colleagues to take all the credit. We acknowledge with gratitude his personal and scientific contributions to our ability to undertake silicone-based science and, for this paper, rename the reaction the Piers–Chojnowski–Rubinsztajn–Kawakami (PCRK) reaction. The metathesis reaction should have his name.

## Sample Availability

Samples of the compounds are not available from the authors.
