# Supplementary material for: When Attempting Chain Extension, Even Without Solvent, It Is Not Possible to Avoid Chojnowski Metathesis Giving D3
Source: Molecules. 2021 Jan 5;26(1):231. doi: 10.3390/molecules26010231 (PMC7795595; doi:10.3390/molecules26010231)
Supplement: Supplementary file 1 [file molecules-26-00231-s001.pdf]

Even Without Solvent It Is Not Possible to Avoid Chojnowski Metathesis Giving D_3

Mengchen Liao,[1] Yang Chen,[1] and Michael A. Brook[1,*]

[1] Department of Chemistry and Chemical Biology, McMaster University, 1280 Main St. W., Hamilton ON Canada L8S 4M1.

* Correspondence: mabrook@mcmaster.ca

Supplementary Material (SM)

Table of Contents

Calculation of polymer molecular weight (M_n) using ^{1}H-NMR 2

'Wet' toluene as the only water source: 3

Characterization of high molecular weight PDMS preparation using hydrolysis (Table 1, Entry 1–6) 4

Characterization for hydrolysis reactions capturing volatiles using a cold trap condition (Table 1, Entry 7,8) 5

Neat (Table 1, Entry 7) 5

50% wt. toluene (Table 1, Entry 8) 7

Monitoring hydrolysis/condensation processes neat (Table 2, Entries 1–9) 9

Characterizations for monitoring hydrolysis process under 50% wt. dry toluene (Table 1, Entry 1–9) 12

Characterizations for 'Living' reaction using hydrolysis of M^HM^H (Table 2, Entry 10) ...14

Figure S1. The proposed structure for low molecular weight silicones 3

Figure S2. ^{29}Si-NMR spectra of the cold-trap reaction using M^HM^H and water in the presence of $B(C_6F_5)_3$ catalyst (neat). 7

Figure S3. ^{29}Si-NMR spectra of the cold-trap reaction using M^HM^H and water in the presence of $B(C_6F_5)_3$ catalyst (50 wt% dry toluene) 9

Figure S4 A: Plot of reaction constituents based on ^{29}Si-NMR (neat). While small differences will exist in the sensitivity of the M vs D units (even in the presence of $Cr(acac)_3$) the presented data represents the Si monomer concentration normalized to 100% B: IR spectra at the same time points. 10

Figure S5 A: Plot of reaction constituents based on ^{29}Si-NMR (with toluene). While small differences will exist in the sensitivity of the M vs D units (even in the presence of

$Cr(acac)_3$) the presented data represents the Si monomer concentration normalized to 100% B: IR spectra at the same time points. 14

Table S1. Summary of D_3, D_4 and D_5 formation in Entries 1–6. Table 1....................5
Table S2 The yield of different compounds formed from hydrolysis of M^HM^H under cold trap condition 5
Table S3 GC-MS data for volatile products collected in cold trap using hydrolysis of tetramethyldisiloxane in neat....................6
Table S4. GC-MS data for volatile products collected in cold trap using hydrolysis of tetramethyldisiloxane in 50% wt. dry toluene....................8

Calculation of polymer molecular weight (M_n) using ^{1}H NMR

Here, we use the 4 min (50% wt. dry toluene) sample as an example (peak integration Si*H*: SiC*H*$_3$=7.65:100) for calculating MW using ^{1}H-NMR.

We assume all the SiH functional groups are at the termini (Figure S1), then the Si*H* peak can be integrated into 2 (one SiH at each end) and if the number of silicon in SiC*H*$_3$ assigned to x (6 hydrogens attached to 1 silicon), we can write the equation as follows:

$$\frac{2}{7.65} = \frac{6x}{100}$$

$$x=4.357$$

The mass of the end group is 59 ($Si(CH_3)_2H$: 28 + 15 × 2 + 1 = 59 g·mol^{-1}), two end group plus one oxygen (not containing in the repeating unit) should be as follows:

$$\text{MW of two SiH end group and one oxygen atom} = 59 \times 2 + 16 = 134 \ \text{g·mol}^{-1}$$

Apart from 2 Si at the end, there are 2 Si left in the repeating unit (MW of the repeating unit $Si(CH_3)_2O$: 28 + 15 × 2 + 16 = 74 g·mol^{-1}) and thus, we can calculate the molecular weight as follows:

$$MW = (x-2) \times 74 + 134 = 308 \approx 310 \ \text{g·mol}^{-1}$$

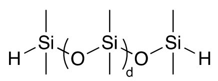

Figure S1. The proposed structure for low-molecular-weight silicones.

"Wet" toluene as the only water source ([OH]/[SiH] = 1, [$B(C_6F_5)_3$]/[SiH] = 0.02 mol%)

To a pre-dried 1.0 L three-neck round-bottomed flask, tetramethyldisiloxane (M^HM^H) (134 g mol^{-1}, 0.23 g, 0.0017 mol) and "wet" toluene (71.62 mL, 466.58 ppm water, 0.0016 mmol of water present) were added, and then capped with a septum with a needle with a bubbler open to atmosphere to balance the pressure. The mixture was stirred for 5–10 minutes prior to the addition of $B(C_6F_5)_3$ stock solution in dry toluene (0.001 M, 0.06 mL, 0.0006 mmol). The $B(C_6F_5)_3$ stock solution was added by Eppendorf pipette into the flask through opening the septa. No obvious bubble formation was observed once catalyst was added. The mixture was stirred at room temperature for 24h and the aliquot was taken after 5h reaction time to run ^{1}H NMR. Finally, the reaction was quenched by alumina for 5h and the residual water droplet was removed by adding sodium sulfate.

5 h reaction time:

^{1}H-NMR (600 MHz, chloroform-*d*): δ 4.71-4.75 (Si*H*, m, 8.35H), 3.76–3.80 ($Si(CH_3)_2H_2$, sep, *J* =4.1 Hz, 2.22H), 1.42 (H_2O, s, 1.42H), 0.09-0.22 (Si-CH_3, m, 100H) ppm.

24 h reaction time:

^{1}H-NMR (600 MHz, chloroform-*d*): δ 4.70–4.74 (Si*H*, m, 8.35H), 3.76-3.79 ($Si(CH_3)_2H_2$, sep, *J* =4.1 Hz, 3.68H), 1.46 (H_2O, s, 2.12H), 0.08–0.21 (Si-CH_3, m, 100H) ppm.

Characterization of high-molecular-weight PDMS preparation using hydrolysis (Table 1, Entry 1–6)

Entry 1: ^{1}H-NMR (600 MHz, chloroform-*d*): δ 1.54 (*H_2*O, s, 4.95H), 0.07-0.17 (Si-C*H_3*, m, 100H) ppm. ^{29}Si NMR (600 MHz, chloroform-*d*, 119 MHz, trace Cr(acac)$_3$): δ -8.27 (*D_3*, s, 2.02Si), −21.50-(-) 22.49 (*D*, m, 100Si) ppm. (GPC, Toluene) M_n = 43,700, M_w = 129,200 g·mol^{-1}, $Đ_M$: 2.97. (Yield: 70.1%).

Entry 2: ^{1}H-NMR (600 MHz, chloroform-*d*): δ 1.55 (*H_2*O, s, 12.18H), 0.07-0.17 (Si-C*H_3*, m, 100H) ppm. ^{29}Si NMR (600 MHz, chloroform-*d*, 119 MHz, trace Cr(acac)$_3$): δ -8.26 (*D_3*, s, 35.50Si), -21.93-(-) 22.16 (*D*, m, 100Si) ppm. (GPC, Toluene) M_n=52,700, M_w=105,400 g mol^{-1}, $Đ_M$: 2.00. (Yield: 71.3%).

Entry 3: ^{1}H-NMR (600 MHz, chloroform-*d*): δ 1.54 (*H_2*O, s, 5.84H), 0.06-0.17 (Si-C*H_3*, m, 100H) ppm. ^{29}Si NMR (600 MHz, chloroform-*d*, 119 MHz, trace Cr(acac)$_3$): δ -8.27 (*D_3*, s, 5.05Si), -19.06 (*D_4*, s, 1.29Si), -21.50-(-) 22.49 (*D*, m, 100Si) ppm. (GPC, Toluene) M_n=41,600, M_w=101,500 g mol^{-1}, $Đ_M$: 2.44. (Yield: 70.6%).

Entry 4: ^{1}H-NMR (600 MHz, chloroform-*d*): δ 1.54 (*H_2*O, s, 1.62H), 0.07–0.17 (Si-C*H_3*, m, 100H) ppm. ^{29}Si NMR (600 MHz, chloroform-*d*, 119 MHz, trace Cr(acac)$_3$): δ -8.29 (*D_3*, s, 3.69Si), -10.36 (s, 1.55Si), -19.06 (*D_4*, s, 2.74Si), −21.50-(-) 22.50 (*D*, m, 100Si) ppm. (GPC, Toluene, before kugelrohr) M_n=20,700, M_w=32,500 g mol^{-1}, $Đ_M$: 1.57, (GPC, Toluene, after kugelrohr) M_n=20,700, M_w=32,500 g mol^{-1}, $Đ_M$: 1.57. (Yield: 79.3%).

Entry 5: ^{1}H-NMR (600 MHz, chloroform-*d*): δ 1.54 (*H_2*O, s, 12.13H), 0.07–0.17 (Si-C*H_3*, m, 100H) ppm. ^{29}Si-NMR (600 MHz, chloroform-*d*, 119 MHz, trace Cr(acac)$_3$): δ -8.26 (*D_3*, s, 11.90Si), -19.06 (*D_4*, s, 2.87Si), −21.50–(-) 22.49 (*D*, m, 100Si) ppm. (GPC, Toluene) M_n = 111,400, M_w = 221,500 g mol^{-1}, $Đ_M$: 1.99. (Yield: 95.6%).

Entry 6: ^{1}H-NMR (600 MHz, chloroform-*d*): δ 1.54 (*H_2*O, s, 2.82H), 0.07-0.17 (Si-C*H_3*, m, 100H) ppm. ^{29}Si-NMR (600 MHz, chloroform-*d*, 119 MHz, trace Cr(acac)$_3$): δ -8.26 (*D_3*, s, 3.07Si), −19.06 (*D_4*, s, 3.06Si), −21.49–(-) 22.48 (*D*, m, 100Si) ppm. (GPC, Toluene) M_n = 152,500, M_w=314,000 g·mol^{-1}, $Đ_M$: 2.06. (Yield: 82.9%).

Table S1. Summary of D_3, D_4 and D_5 formation in Entries 1-6. Table 1

Entry	End group	D_3 (%)	DM^{OH}	D_4 (%)	D_5 (%)	D (%)[a]
		−8.3 ppm	−10.4 ppm	−19.1 ppm	−21.5 ppm	−22ppm
1	SiOH	8.45	0	2.54	9.46	79.55
2	SiOH	12.93	0	0	10.78	76.29
3	SiOH	4.21	0	1.82	8.21	85.76
4	SiOH	3.74	0.88	2.27	3.82	89.29
5	SiOH	8.95	0	2.04	8.38	80.63
6	SiOH	3.88	0	3.38	5.4	87.34

[a] All peaks appeared at D unit are normalized to 100.

Characterization for hydrolysis reactions capturing volatiles using a cold trap condition (Table 1, Entry 7,8)

Table S2. The yield of different compounds formed from hydrolysis of M^HM^H under cold trap conditions.

Conditions	Actual starting material mass (g)[a]	D_3 (%)[b]	D_4 (%)	D_5 (%)	Polymeric (%)	M^HDM^H (%)	M^HDM^{OH} (%)	Me_2SiH_2 (%)
Neat	10.3236	13.32	0.76	2.32	67.96	1.11	0.59	11.31
50 wt% dry toluene	10.8532	33.01	2.81	10.23	49.89	0.43	0.25	2.95

[a] Calculation deduct the mass of the unreacted M^HM^H collected in the cold trap condenser. [b] The ratio of each component was identified by ^{29}Si-NMR.

Neat (Table 1, Entry 7)

To a pre-dried 100.0 mL round-bottomed flask tetramethyldisiloxane (M^HM^H) (134 g·mol^{-1}, 10.38 g, 77.5 mmol) with distilled water (18 g·mol^{-1}, 1.34 mL, 74.4 mmol) was added. To the mixture $B(C_6F_5)_3$ stock solution in dry toluene (0.1 M, 0.298 mL, 0.0298 mmol) was added. The cold trap condenser was submerged in dry ice/acetone with the addition of acetone and was directly connected to the round-bottomed flask through 6 mm O.D. Pyrex tubing. The mixture in the round bottle flask was stirred at room temperature for 3h and the reaction was quenched by alumina and the residual water droplet was removed by adding

sodium sulfate with 5h quenching time. Before the work up procedures, the mixtures in the round bottle flask were weighed and characterized by ^{1}H, ^{29}Si-NMR (SR) followed by filtration through Celite under reduced pressure.

Cold trap: ^{1}H-NMR (600 MHz, chloroform-*d*): δ 4.67-4.70 (Si*H*, sep, *J* =2.8 Hz, 13.99H), 3.74-3.77 ($Si(CH_3)_2H_2$, sep, *J* =4.1 Hz, 1.89H), 1.52 (H_2O, s, 0.41H), 0.07-0.20 ($Si\text{-}CH_3$, m, 100H) ppm. ^{29}Si NMR (600 MHz, chloroform-*d*, 119 MHz, trace $Cr(acac)_3$): δ -4.49 (M^HM^H, s, 21.91Si), -6.67 (M^HDM^H, s, 1.81Si), -8.35 (D_3, s, 1.00Si), -17.79 (M^HDM^{OH}, s, 0.96Si), -37.82 ($Me_2\underline{Si}H_2$, s, 18.38Si) ppm. Total Mass in trap: 2.798 g, 23.9%mass.

Polymeric material: ^{1}H-NMR (600 MHz, chloroform-*d*): δ 4.63 (Si*H*, s, 0.04H), 1.54 (H_2O, s, 1.90H), 0.06-0.17 ($Si\text{-}CH_3$, m, 100H) ppm. ^{29}Si-NMR (600 MHz, chloroform-*d*, 119 MHz, trace $Cr(acac)_3$): δ -8.28 (D_3, s, 18.70Si), -19.07 (D_4, s, 1.12Si), -21.50 (D_5, S, 3.41Si), -21.95- (-)22.17 (*D*, m, 100Si) ppm. (GPC, Toluene) M_n = 20,500, M_w = 37,400 g·mol^{-1}, $Đ_M$:1.82. Total Mass in reaction vessel: 8.646 g, 73.8%mass (total recovery, 97.7%).

Table S3. GC-MS data for volatile products collected in cold trap using hydrolysis of tetramethyldisiloxane neat.

Compound	Structures	Elution time (min)	MW (g·mol^{-1})	GC-MS relative ratio (%)	Yield (%)[a]
M^HM^H	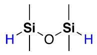	1.6	134.32	93.8	23.9
M^HDM^H	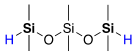	2.9	208.48	6.2	

[a] Yield was measured as mass balance before and after the reaction in the cold trap.

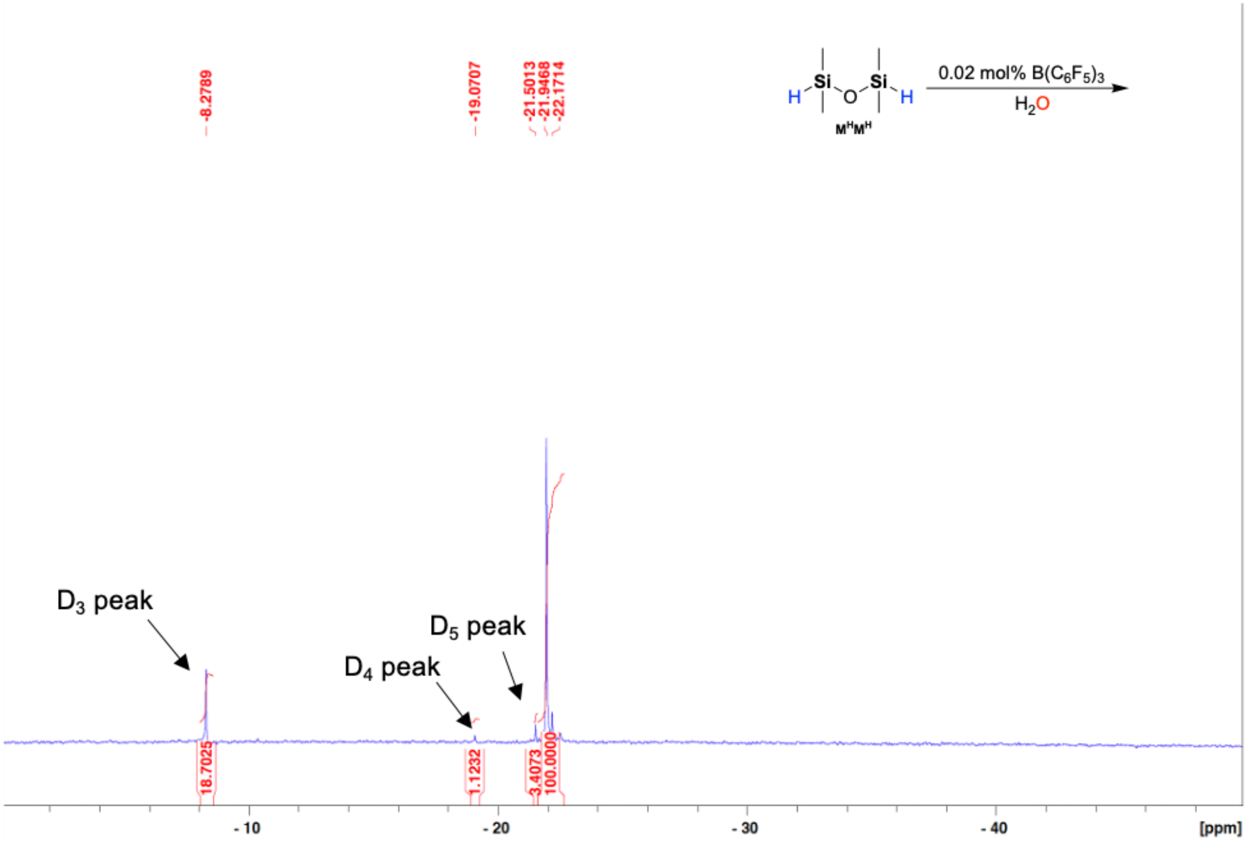

Figure S2. ^{29}Si NMR spectra of the cold-trap reaction using M^HM^H and water in the presence of $B(C_6F_5)_3$ catalyst (neat).

50% wt. toluene (Table 1, Entry 8)

To a pre-dried 100.0 ml round-bottomed flask, tetramethyldisiloxane (M^HM^H) (134 g·mol^{-1}, 10.32 g, 77.0 mmol) with distilled water (18 g·mol^{-1}, 1.34 mL, 74.4 mmol) and dry toluene (6.73 mL, 5.83 g, m_{MHMH}(g)/$V_{toluene}$ (mL)=1.53, m_{MHMH}+m_{H2O} = 11.66 g) were added. To the mixture $B(C_6F_5)_3$ stock solution in dry toluene (0.1 M, 0.298 mL, 0.0298 mmol) was added. The cold trap condenser was submerged in dry ice/acetone with the addition of acetone and was directly connected to the round-bottomed flask through 6 mm O.D. Pyrex tubing. The mixture in the round bottle flask was stirred at room temperature for 3h and the reaction was quenched by alumina and the residual water droplet was removed by adding sodium sulfate with 5h quenching time. Before the work up procedures, the mixtures in the round bottle flask were weighed and characterized by ^1H-, ^{29}Si-NMR (SR) followed by filtration through Celite under reduced pressure.

Cold trap: ^{1}H-NMR (600 MHz, chloroform-*d*): δ 4.68-4.71 (Si*H*, sep, *J* =2.8 Hz, 10.44H), 3.74-3.77 ($Si(CH_3)_2H_2$, sep, *J* =4.1 Hz, 8.42H), 1.53 (H_2O, s, 0.32H), 0.07-0.20 ($Si\text{-}CH_3$, m, 100H) ppm. ^{29}Si-NMR (600 MHz, chloroform-*d*, 119 MHz, trace $Cr(acac)_3$): δ -4.49 (M^HM^H, s, 42.30Si), -6.66 (M^HDM^H, s, 2.44Si), -8.33 (D_3, s, 1.00Si), -17.77 (M^HDM^{OH}, s, 1.40Si), -37.82 ($Me_2\underline{Si}H_2$, s, 16.67Si) ppm. Total Mass in trap: 1.224 g, 10.5%mass.

Polymeric material: ^{1}H-NMR (600 MHz, chloroform-*d*): δ 4.70 (Si*H*, s, 0.01H), 1.50 (H_2O, s, 0.42H), 0.08-0.18 ($Si\text{-}CH_3$, m, 100H) ppm. ^{29}Si-NMR (600 MHz, chloroform-*d*, 119 MHz, trace $Cr(acac)_3$): δ -8.26 (D_3, s, 65.81Si), -19.02 (D_4, s, 5.63Si), -21.44 (D_5, s, 20.50), -22.11- (-) 22.43 (*D*, m, 100Si) ppm. (GPC, Toluene) M_n=40,000, M_w=63,100 g·mol^{-1}, $Đ_M$: 1.58. Total Mass in reaction vessel: 10.393 g, 89.1% mass (total recovery, 99.6%).

Table S4. GC-MS data for volatile products collected in cold trap using hydrolysis of tetramethyldisiloxane in 50% wt. dry toluene.

Compound	Structures	Elution time (min)	MW (g mol^{-1})	GC-MS relative ratio (%)	Yield (%)[a]
M^HM^H	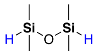	1.6	134.32	85.6	
M^HDM^H	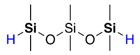	2.9	208.48	11	10.5
D_3	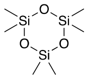	4.7	222.46	3.4	

[a] Yield was measured as mass balance before and after the reaction in the cold trap.

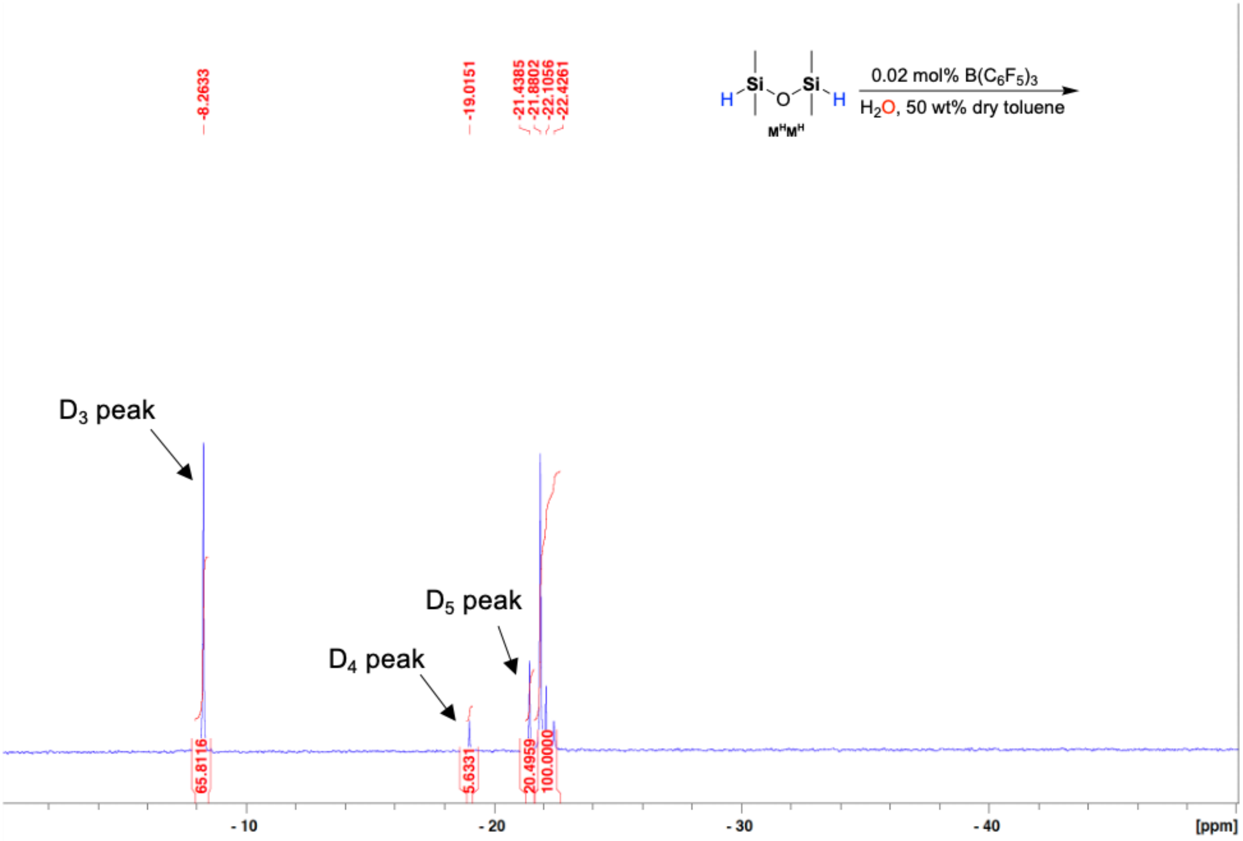

Figure S3. ^{29}Si-NMR spectra of the cold-trap reaction using M^HM^H and water in the presence of $B(C_6F_5)_3$ catalyst (50 wt% dry toluene).

Monitoring hydrolysis/condensation processes neat (Table 2, Entries 1-9)

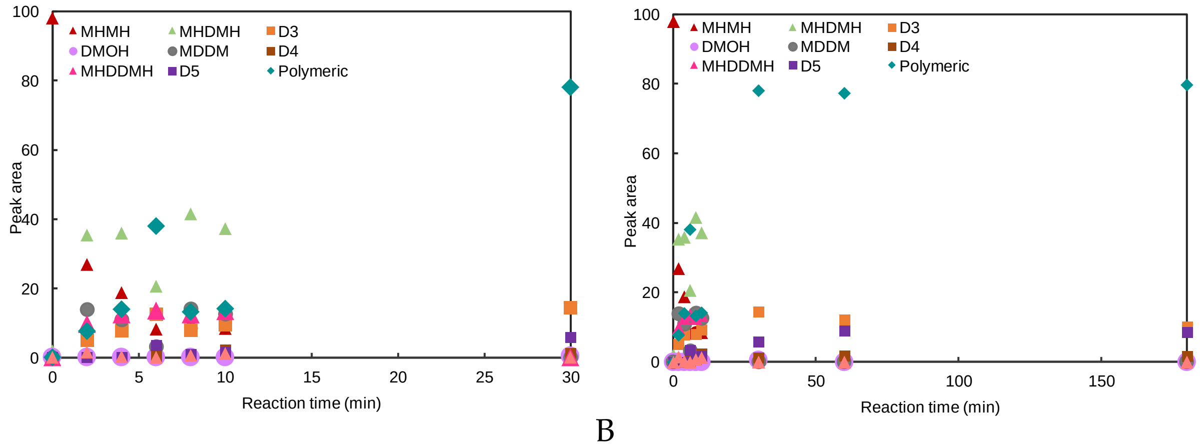

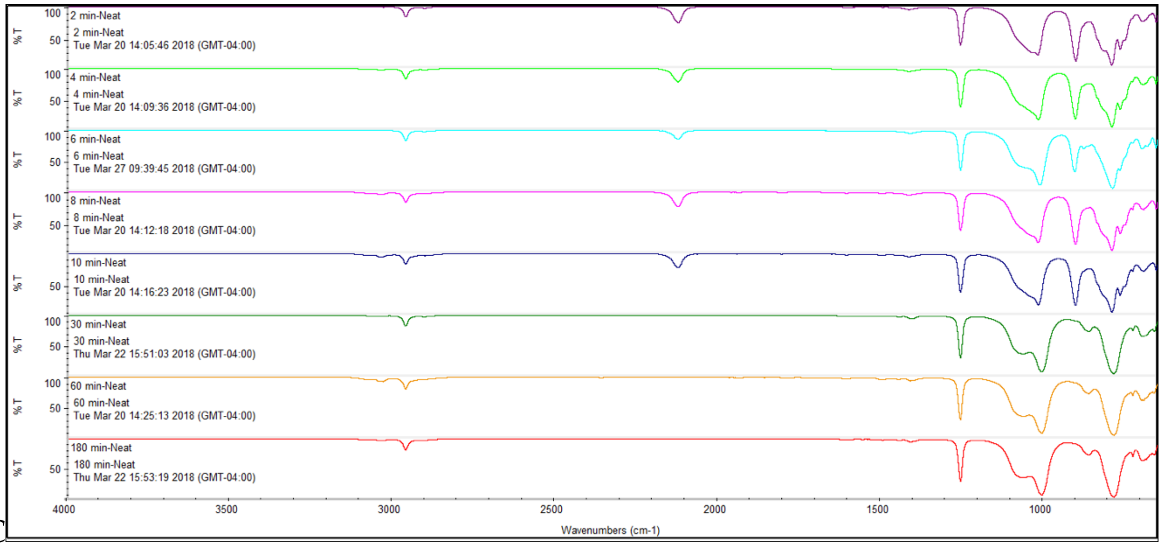

C

Figure S4. (A) Plot of reaction constituents based on ^{29}Si NMR (neat). While small differences will exist in the sensitivity of the M vs D units (even in the presence of Cr(acac)$_3$) the presented data represent the Si monomer concentration normalized to 100% (first 30 min). (B) Same plot to 180 min. (C) IR spectra at the same time points.

0 min: ^{1}H-NMR (600 MHz, chloroform-*d*): δ 4.67-4.68 (Si*H*, sep, *J* = 2.8 Hz, 14.76H), 1.54 (H_2O, s, 0.26H), 0.19 (Si-CH_3, d, *J* =2.8 Hz, 100H) ppm. ^{29}Si-NMR (600 MHz, chloroform-*d*, 119 MHz, trace Cr(acac)$_3$): δ -4.45 (M^HM^H, s, 98.04Si), -6.63 (M^HDM^H, s, 1.96Si) ppm.

2 min: ^{1}H-NMR (600 MHz, chloroform-*d*): δ 4.67-4.71 (Si*H*, m, 9.59H), 3.74-3.77 (Si(CH$_3$)$_2$$H_2$, sep, *J* =3.9 Hz, 0.09H), 1.53 (H_2O, s, 0.55H), 0.07-0.20 (Si-CH_3, m, 100H) ppm. ^{29}Si-NMR (600 MHz, chloroform-*d*, 119 MHz, trace Cr(acac)$_3$): δ -4.49 (M^HM^H, s, 26.80 Si), -6.66-(-)6.88 (M^HDM^H, m, 35.36Si), -8.33 (D_3, s, 5.13Si), -17.76 (M^H*D*M^{OH}, s, 13.77Si), -19.72-(-) 19.83 (M^H*DD*M^H, 9.74), -21.69-(-)21.78 (*D*, m, 7.73Si), -37.85 (Me$_2$*Si*H$_2$, s, 1.46Si) ppm.

4 min: ^{1}H-NMR (600 MHz, chloroform-*d*): δ 4.67-4.71 (Si*H*, m, 7.87H), 3.74-3.77 (Si(CH$_3$)$_2$$H_2$, sep, *J* =4.1 Hz, 0.12H), 1.53 (H_2O, s, 0.46H), 0.07-0.20 (Si-CH_3, m, 100H) ppm. ^{29}Si-NMR (600 MHz, chloroform-*d*, 119 MHz, trace Cr(acac)$_3$): δ -4.49 (M^HM^H, s, 18.80 Si), -6.67-(-)6.90 (M^HDM^H, m, 35.89Si), -8.33 (D_3, s, 7.88Si), -17.76 (M^H*D*M^{OH}, s, 10.85Si), -19.83-(-) 19.87 (M^H*DD*M^H, 12.40), -21.70-(-)21.91 (*D*, m, 14.01Si), -37.84 (Me$_2$*Si*H$_2$, s, 0.18Si) ppm.

6 min: ^{1}H-NMR (600 MHz, chloroform-*d*): δ 4.67-4.71 (Si*H*, m, 4.58H), 3.74-3.77 ($Si(CH_3)_2H_2$, sep, *J* =4.2 Hz, 0.02H), 1.54 (H_2O, s, 0.44H), 0.07-0.20 (Si-CH_3, m, 100H) ppm. ^{29}Si-NMR (600 MHz, chloroform-*d*, 119 MHz, trace $Cr(acac)_3$): δ -4.48 (M^HM^H, s, 8.18 Si), -6.66-(-)6.90 (M^HDM^H, m, 20.58Si), -8.32 (D_3, s, 12.68Si), -17.75 (M^HDM^{OH}, s, 3.17Si), -19.10 (D_4, s, 0.39Si), -19.72-(-) 19.88 (M^HDDM^H, 13.51), -21.53 (D_5, s, 3.49Si), -21.69-(-)22.52 (*D*, m, 38Si) ppm.

8 min: ^{1}H-NMR (600 MHz, chloroform-*d*): δ 4.67-4.71 (Si*H*, m, 7.51H), 3.73-3.77 ($Si(CH_3)_2H_2$, sep, *J* =4.1 Hz, 0.28H), 1.53 (H_2O, s, 0.41H), 0.07-0.20 (Si-CH_3, m, 100H) ppm. ^{29}Si-NMR (600 MHz, chloroform-*d*, 119 MHz, trace $Cr(acac)_3$): δ -4.49 (M^HM^H, s, 8.40 Si), -6.66-(-)6.90 (M^HDM^H, m, 41.50Si), -8.32 (D_3, s, 8.03Si), -17.75 (M^HDM^{OH}, s, 14.11Si), -19.10 (D_4, s, 0.75Si), -19.72-(-) 19.86 (M^HDDM^H, 12.45), -21.53 (D_5, s, 1.00Si), -21.69-(-)22.20 (*D*, m, 13.13Si), -37.84 (Me_2SiH_2, s, 0.63Si) ppm.

10 min: ^{1}H-NMR (600 MHz, chloroform-*d*): δ 4.67-4.71 (Si*H*, m, 7.12H), 3.73-3.77 ($Si(CH_3)_2H_2$, sep, *J* =4.1 Hz, 0.19H), 1.53 (H_2O, s, 0.38H), 0.07-0.20 (Si-CH_3, m, 100H) ppm. ^{29}Si-NMR (600 MHz, chloroform-*d*, 119 MHz, trace $Cr(acac)_3$): δ -4.49 (M^HM^H, s, 8.35 Si), -6.66-(-)6.90 (M^HDM^H, m, 37.22Si), -8.33 (D_3, s, 9.40Si), -17.75 (M^HDM^{OH}, s, 12.47Si), -19.10 (D_4, s, 2.25Si), -19.72-(-) 19.86 (M^HDDM^H, 13.33), -21.53 (D_5, s, 1.58Si), -21.78-(-)21.82 (*D*, m, 14.15Si), -37.84 (Me_2SiH_2, s, 1.25Si) ppm.

30 min: ^{1}H-NMR (600 MHz, chloroform-*d*): δ 4.69-4.71 (Si*H*, m, 0.06H), 1.56 (H_2O, s, 7.49H), 0.06-0.17 (Si-CH_3, m, 100H) ppm. ^{29}Si-NMR (600 MHz, chloroform-*d*, 119 MHz, trace $Cr(acac)_3$): δ -8.27 (D_3, s, 14.52Si), -10.38 ($M^{OH}D$, 0.44Si), -19.07 (D_4, s, 1.20Si), -21.50 (D_5, s, 5.84Si), -21.94-(-)22.49 (*D*, m, 78.00Si) ppm. (GPC, Toluene) M_n=21,600, M_w=54,000 g mol^{-1}, $Đ_M$: 2.50.

60 min: ^{1}H-NMR (600 MHz, chloroform-*d*): δ 4.62 (Si*H*, s, 0.03H), 1.56 (H_2O, s, 4.39H), 0.07-0.17 (Si-CH_3, m, 100H) ppm. ^{29}Si-NMR (600 MHz, chloroform-*d*, 119 MHz, trace $Cr(acac)_3$): δ -8.28 (D_3, s, 12.04Si), -19.07 (D_4, s, 1.77Si), -21.50 (D_5, s, 8.89Si), -21.95-(-)22.49 (*D*, m, 77.30Si) ppm. (GPC, Toluene) M_n=21,800, M_w=63,000 g mol^{-1}, $Đ_M$: 2.90.

180 min: ^{1}H-NMR (600 MHz, chloroform-*d*): δ 1.53 (H_2O, s, 0.57H), 0.07-0.17 (Si-CH_3, m, 100H) ppm. ^{29}Si-NMR (600 MHz, chloroform-*d*, 119 MHz, trace Cr(acac)$_3$): δ -8.28 (D_3, s, 10.07Si), -19.07 (D_4, s, 1.64Si), -21.51 (D_5, s, 8.60Si), -21.95-(-)22.50 (*D*, m, 79.69Si) ppm. (GPC, Toluene) M_n=31,200, M_w=82,900 g mol^{-1}, $Đ_M$: 2.66.

Characterizations for monitoring hydrolysis process under 50% wt. dry toluene (Table 2, Entry 1-9)

0 min: ^{1}H-NMR (600 MHz, chloroform-*d*): δ 4.67-4.68 (Si*H*, sep, *J* = 2.8 Hz, 14.76H), 1.54 (H_2O, s, 0.26H), 0.19 (Si-CH_3, d, *J* =2.8 Hz, 100H) ppm. ^{29}Si-NMR (600 MHz, chloroform-*d*, 119 MHz, trace Cr(acac)$_3$): δ -4.45 (M^HM^H, s, 98.04Si), -6.63 (M^HDM^H, s, 1.96Si) ppm.

2 min: ^{1}H-NMR (600 MHz, chloroform-*d*): δ 4.68-4.72 (Si*H*, m, 9.43H), 3.75-3.77 (Si(CH$_3$)$_2$$H_2$, sep, *J* =4.1 Hz, 0.10H), 1.52 (H_2O, s, 1.25H), 0.07-0.20 (Si-CH_3, m, 100H) ppm. ^{29}Si-NMR (600 MHz, chloroform-*d*, 119 MHz, trace Cr(acac)$_3$): δ -4.41 (M^HM^H, s, 24.18 Si), -6.58-(-)6.80 (M^HDM^H, m, 36.67Si), -8.28 (D_3, s, 8.09Si), -17.70 (M^H*D*M^{OH}, s, 14.34Si), -19.66-(-) 19.76 (M^H*DD*M^H, 9.40), -21.63-(-)21.72 (*D*, m, 6.00Si), -37.85 (Me$_2$*Si*H$_2$, s, 1.32Si) ppm.

4 min: ^{1}H-NMR (600 MHz, chloroform-*d*): δ 4.68-4.72 (Si*H*, m, 7.65H), 3.74-3.78 (Si(CH$_3$)$_2$$H_2$, sep, *J* =4.1 Hz, 0.33H), 1.52 (H_2O, s, 2.60H), 0.07-0.21 (Si-CH_3, m, 100H) ppm. ^{29}Si-NMR (600 MHz, chloroform-*d*, 119 MHz, trace Cr(acac)$_3$): δ -4.38 (M^HM^H, s, 10.58 Si), -6.55-(-)6.77 (M^HDM^H, m, 38.57Si), -8.23 (D_3, s, 14.40Si), -17.65 (M^H*D*M^{OH}, s, 12.86Si), -19.00 (D_4, s, 0.72Si), -19.61-(-) 19.72 (M^H*DD*M^H, 11.89), -21.58-(-)21.67 (*D*, m, 9.90Si), -37.73 (Me$_2$*Si*H$_2$, s, 1.08Si) ppm.

6 min: ^{1}H-NMR (600 MHz, chloroform-*d*): δ 4.68-4.72 (Si*H*, m, 7.18H), 3.74-3.77 (Si(CH$_3$)$_2$$H_2$, sep, *J* =4.1 Hz, 0.50H), 1.52 (H_2O, s, 1.39H), 0.06-0.21 (Si-CH_3, m, 100H) ppm. ^{29}Si-NMR (600 MHz, chloroform-*d*, 119 MHz, trace Cr(acac)$_3$): δ -4.42 (M^HM^H, s, 7.09 Si), -6.59-(-)6.83 (M^HDM^H, m, 39.28Si), -8.28 (D_3, s, 14.30Si), -17.70 (M^H*D*M^{OH}, s, 13.11Si), -19.04 (D_4, s, 1.23Si), -19.66-(-) 19.80 (M^H*DD*M^H, 12.37), -21.46 (D_5, s, 1.11Si), -21.63-(-)22.13 (*D*, m, 10.70Si), -37.77 (Me$_2$*Si*H$_2$, s, 0.81Si) ppm.

8 min: ^{1}H-NMR (600 MHz, chloroform-*d*): δ 4.68-4.72 (Si*H*, m, 5.38H), 3.74-3.78 ($Si(CH_3)_2H_2$, sep, *J* =4.1 Hz, 0.30H), 1.52 (H_2O, s, 1.06H), 0.07-0.21 (Si-CH_3, m, 100H) ppm. ^{29}Si-NMR (600 MHz, chloroform-*d*, 119 MHz, trace Cr(acac)$_3$): δ -4.42 (M^HM^H, s, 3.92 Si), -6.59-(-)6.82 (M^HDM^H, m, 30.69Si), -8.28 (D_3, s, 21.04Si), -17.70 (M^H*D*M^{OH}, s, 7.85Si), -19.04 (D_4, s, 1.66Si), -19.66-(-) 19.81 (M^H*DD*M^H, 13.59), -21.47 (D_5, s, 1.91Si), -21.63-(-)22.14 (*D*, m, 19.34Si) ppm.

10 min: ^{1}H-NMR (600 MHz, chloroform-*d*): δ 4.68-4.73 (Si*H*, m, 2.36H), 3.75-3.78 ($Si(CH_3)_2H_2$, sep, *J* =4.2 Hz, 0.03H), 1.52 (H_2O, s, 0.98H), 0.07-0.20 (Si-CH_3, m, 100H) ppm. ^{29}Si-NMR (600 MHz, chloroform-*d*, 119 MHz, trace Cr(acac)$_3$): δ -4.42 (M^HM^H, s, 0.71 Si), -6.59-(-)6.84 (M^HDM^H, m, 14.99Si), -8.29 (D_3, s, 30.65Si), -17.71 (M^H*D*M^{OH}, s, 2.11Si), -19.05 (D_4, s, 2.88Si), -19.83 (M^H*DD*M^H, 9.46), -21.48 (D_5, s, 6.29Si), -21.64-(-)22.47 (*D*, m, 32.91Si) ppm.

30 min: ^{1}H-NMR (600 MHz, chloroform-*d*): δ 1.53 (H_2O, s, 1.89H), 0.07-0.19 (Si-CH_3, m, 100H) ppm. ^{29}Si-NMR (600 MHz, chloroform-*d*, 119 MHz, trace Cr(acac)$_3$): δ -8.28 (D_3, s, 29.04Si), -19.05 (D_4, s, 3.27Si), -21.47 (D_5, s, 13.24Si), -21.92-(-)22.46 (*D*, m, 54.45Si) ppm. (GPC, Toluene) M_n=39,000, M_w=69,700 g mol^{-1}, $Đ_M$:1.79.

60 min: ^{1}H-NMR (600 MHz, chloroform-*d*): δ 1.52 (H_2O, s, 1.42H), 0.07-0.19 (Si-CH_3, m, 100H) ppm. ^{29}Si-NMR (600 MHz, chloroform-*d*, 119 MHz, trace Cr(acac)$_3$): δ -8.27 (D_3, s, 29.72Si), -19.04 (D_4, s, 3.58Si), -21.47 (D_5, s, 13.45Si), -21.91-(-)22.46 (*D*, m, 53.25Si) ppm. (GPC, Toluene) M_n=45,300, M_w=97,100 g mol^{-1}, $Đ_M$:2.14.

180 min: ^{1}H-NMR (600 MHz, chloroform-*d*): δ 1.52 (H_2O, s, 1.09H), 0.08-0.18 (Si-CH_3, m, 100H) ppm. ^{29}Si-NMR (600 MHz, chloroform-*d*, 119 MHz, trace Cr(acac)$_3$): δ -8.28 (D_3, s, 28.44Si), -19.06 (D_4, s, 2.30Si), -21.48 (D_5, s, 13.99Si), -21.93-(-)22.47 (*D*, m, 55.27Si) ppm. (GPC, Toluene) M_n=45,500, M_w=79,900 g mol^{-1}, $Đ_M$:1.76.

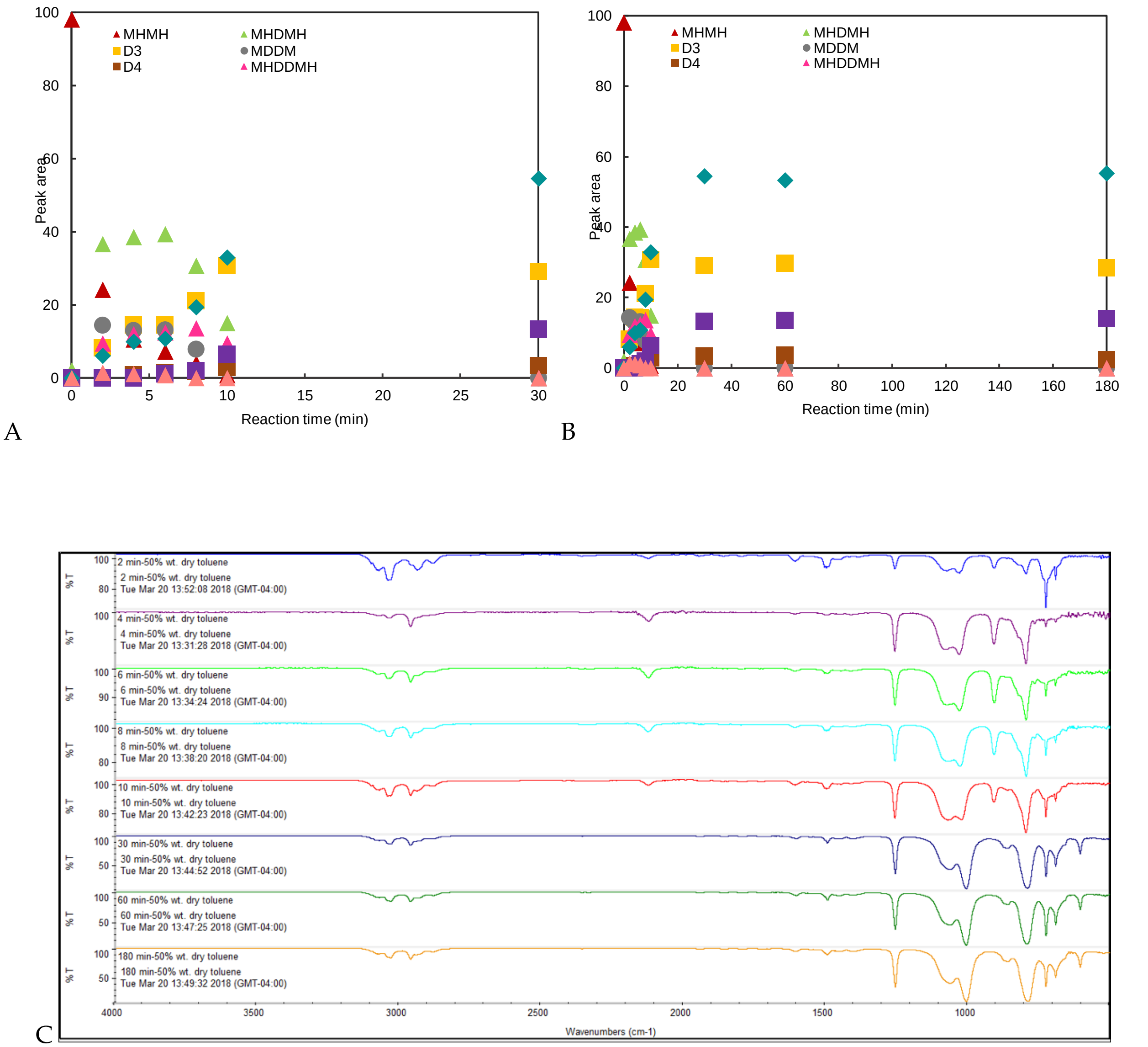

Figure S5. (A) Plot of reaction constituents based on ^{29}Si NMR (with toluene). While small differences will exist in the sensitivity of the M vs D units (even in the presence of Cr(acac)$_3$) the presented data represent the Si monomer concentration normalized to 100%; first 30 min. (B) To 180 min. (C) IR spectra at the same time points.

Characterizations for 'Living' reaction using hydrolysis of M^HM^H (Table 2, Entry 10)

Entry 10: ^{1}H-NMR (600 MHz, chloroform-*d*): δ 1.53 (H_2O, s, 8.61H), 0.07-0.17 (Si-CH_3, m, 100H) ppm. ^{29}Si-NMR (600 MHz, chloroform-*d*, 119 MHz, trace Cr(acac)$_3$): δ -21.95 (*D*, m, 100Si) ppm. (GPC, Toluene) M_n=41,900, M_w=137,000 g mol^{-1}, $Đ_M$: 3.27.
